# Is *Consent-GPT* valid? Public attitudes to generative AI use in surgical consent

**DOI:** 10.1007/s00146-025-02644-9

**Published:** 2025-10-09

**Authors:** Jemima Winifred Allen, Ivar Rodríguez Hannikainen, Julian Savulescu, Dominic Wilkinson, Brian David Earp

**Affiliations:** 1 Monash University, Melbourne, Australia; 2 University of Oxford, Oxford, United Kingdom; 3 University of Granada, Granada, Spain; 4 National University of Singapore, Singapore, Singapore; 5 John Radcliffe Hospital, Oxford, United Kingdom; 6 Melbourne, Australia

**Keywords:** Artificial intelligence in healthcare, Consent delegation, Empirical bioethics, Informed consent, Large language models, Medical ethics

## Abstract

Healthcare systems often delegate surgical consent-seeking to members of the treating team other than the surgeon (e.g., junior doctors in the UK and Australia). Yet, little is known about public attitudes toward this practice compared to emerging AI-supported options. This first large-scale empirical study examines how laypeople evaluate the validity and liability risks of using an AI-supported surgical consent system (*Consent-GPT*). We randomly assigned 376 UK participants (demographically representative for age, ethnicity, and gender) to evaluate identical transcripts of surgical consent interviews framed as being conducted by either *Consent-GPT*, a junior doctor, or the treating surgeon. Participants broadly agreed that AI-supported consent was valid (87.6% agreement), but rated it significantly lower than consent sought solely by human clinicians (treating surgeon: 97.6% agreement; junior doctor: 96.2%). Participants expressed substantially lower satisfaction with AI-supported consent compared to human-only processes (*Consent-GPT*: 59.5% satisfied; treating surgeon 96.8%; junior doctor: 93.1%), despite identical consent interactions (i.e., the same informational content and display format). Regarding justification to sue the hospital following a complication, participants were slightly more inclined to support legal action in response to AI-supported consent than human-only consent. However, the strongest predictor was proper risk disclosure, not the consent-seeking agent. As AI integration in healthcare accelerates, these results highlight critical considerations for implementation strategies, suggesting that a hybrid approach to consent delegation that leverages AI’s information sharing capabilities while preserving meaningful human engagement may be more acceptable to patients than an otherwise identical process with relatively less human-to-human interaction.

## 1 Introduction

Informed consent is foundational to medical ethics and good clinical practice. But while obtaining valid consent is usually necessary for respecting patient autonomy, ensuring that patients are adequately informed to provide such consent can be difficult and time-consuming, placing a strain on limited healthcare resources.

To reduce time and energy costs for senior clinicians, it is common in some jurisdictions for part of the consent-seeking process to be delegated to another member of the surgical team (Aasen et al. 2020), although the treating surgeon remains ultimately responsible (The Royal College of Surgeons 2018). Such (partial) consent delegation typically involves junior doctors: qualified medical professionals who have not completed specialty training (Wood et al. 2016; Atkin et al. 2022).^1^

Could similar partial delegation (see Box 1 for clarification) to an artificially intelligent (AI) system further reduce strain on human clinicians? This idea is now being explored in earnest, with a particular focus on large language models (LLMs) given their ability to engage in naturalistic conversation (Aydin et al. 2023; Xiao et al. 2023; Savage et al. 2024; Shi et al. 2023). According to some proposals, an AI-assisted consent system (which we have termed “Consent-GPT;” see Allen et al. 2024a, 2025) should incorporate domain-specific knowledge, including procedural information, risk stratification, and evidence-based clinical guidelines (Barnes et al. 2025). They could also include built-in prompts to ensure legal requirements are met; for instance, automatically asking questions to elicit a patient’s so-called “material” risks (i.e., risks they would find relevant or “material” to their decision), or prompting discussion of alternative treatment options.

In addition to potentially freeing up clinician time and energy for more targeted or complex conversations (i.e., once the routine, legally required information has been initially explained by the AI), AI-supported consent might also partly address limitations of existing human-only or static written consent processes. For example, they could provide more systematic or up-to-date information, while also reducing time pressure on patients—who could consult Consent-GPT outside of clinic hours, possibly through a secure phone app or web link interface—and even boost patient comprehension and engagement (Xiao et al. 2023). Unlike traditional printed consent forms, LLMs allow for dynamic, personalised dialogue, and can immediately clarify information in response to user questions, adapting explanations as needed to individual comprehension levels or even translating information into the user’s native tongue. Additionally, LLMs can provide detailed documentation of an entire consent conversation, creating a comprehensive record of information discussed and patient response.

Of course, LLMs have limitations of their own, including the well-known potential for “hallucinations” (i.e., confidently providing plausible but factually incorrect information) (González-Corbelle et al. 2022), which some research suggests it may be impossible to eliminate completely due to the way generative AI works (Banerjee et al. 2025). Thus, there must always be robust mechanisms of human oversight (Vasey et al. 2022), which in turn might reduce the extent to which AI systems free up human time and energy. There are also questions about how AI systems could meet legal requirements for tailored risk communication and discussion of treatment alternatives specific to each patient’s circumstances.^2^ These and other relevant limitations would need to be addressed prior to the safe implementation of LLMs in clinical practice (Savulescu et al. 2024).

Current proposals for such future, potential implementation range from using LLMs to assist only in providing information and answering patient questions (e.g., Allen et al. 2024b), to handling the bulk of the consent interaction, including the initial discussion of the procedure, its risks and benefits, through to assessment of the patient’s understanding and recording the patient’s final decision (e.g., Allen et al. 2024b). Between these extremes lies a *hybrid* approach, whereby LLMs would aid or enhance the informed consent process by providing systematic information in a user-friendly manner, explaining or elaborating on details in response to patients’ questions, and documenting the entire exchange, while nevertheless preserving meaningful human interaction for key aspects (e.g., performing capacity assessments, confirming understanding) (Allen et al. 2024a).

Early trials of LLMs in consent processes have shown promising results for patient engagement and satisfaction (Aydin et al. 2023; Xiao et al. 2023). LLMs also seem to enable efficiency gains, with one study finding consent was completed 11 days faster with an LLM-supported approach than a human-only one (Savage et al. 2024). However, such studies have primarily focused on technical feasibility and user experience. There remains limited research examining public attitudes toward such approaches, including moral evaluations of the validity of AI-supported consent or its perceived implications for liability (e.g., healthcare system responsibility for error). And yet, this understanding could be crucial as healthcare organisations consider expanding AI’s role in patient interactions.^3^

The current study employs an experimental bioethics approach (Earp et al. 2021) to examine how the type of agent involved in obtaining consent affects public perceptions of consent validity and justification for legal action. We used the contrastive vignette technique (see Reiner 2019; Lewis et al. 2017) to isolate the specific effect of the consent-seeking agent while keeping all other variables constant within the constraints of basic realism/ecological validity. This experimental approach allows us to control the content of the consent conversation (including the specific risks discussed, questions asked, and information provided) while varying only whether this exchange was ostensibly conducted by an AI system, junior doctor, or treating surgeon.^4^

By systematically comparing public responses to the same consent conversations, this study aims to provide crucial insights into the acceptability and perceived validity of AI involvement in medical consent. These findings will help inform the development of ethical frameworks and implementation strategies for AI systems in healthcare consent processes, while identifying potential barriers to public trust and acceptance.

### 1.2 Background & scope

To understand our design choices, study motivation, and interpretation of results, it is necessary for readers to have a shared understanding of certain background information about the nature and function of informed consent. As recognized in medical ethics, law, and clinical practice guidelines, valid informed consent involves more than the simple act of signing a written form (Beauchamp and Childress 2019; Pugh 2020; Hurd 1996; Dougherty 2021). Instead, such consent must meet several criteria: (i) the patient must have decision-making capacity, (ii) be informed about relevant information, (iii) understand this information, (iv) act voluntarily and free from coercion, and (v) be able to communicate their decision (Beauchamp and Childress 2019). The consent-seeking process, particularly for more involved interventions such as surgery, typically involves multiple stages, from initial discussion with the clinical team to final confirmation before the procedure (Leclercq et al. 2010). Professional guidelines emphasize that this process should begin well in advance of surgery to allow patients sufficient time to make properly informed decisions (The Royal College of Surgeons, (The 2018)).

Until recently, legal requirements for informed consent in the UK relied on doctors’ professional judgement to determine what information should be disclosed to patients [known as the *Bolam* test; (Bolam v Friern Hospital Management Committee, 1957)]. Following the landmark ruling in Montgomery v Lanarkshire Health Board (2015), it is no longer seen as sufficient for doctors to provide patients with standardised information or to rely on their own judgment about what should be disclosed. Instead, legally, they must engage in a discussion with patients to identify which risks are *material* to them given the patient’s unique circumstances and values (i.e., the ‘particular patient standard’).

However, current delegation practices in countries like the UK and Australia may not fully meet legal standards, as junior doctors frequently lack adequate training in or knowledge of specific procedures (Propst et al. 2019; Heaney et al. 2019; Atkin et al. 2022; Smith et al. 2016). They may also sometimes fail to discuss key elements of the consent process (e.g., patients’ treatment goals, different management options, and associated risks and benefits) (Atkin et al. 2022; Gardner and AbdelFattah 2017). Studies suggest that patient consent is commonly sought the morning of, or even moments before, surgery (Wood et al. 2016), with nearly half (49%) of respondents to a survey of 550 UK surgeons completing consent discussions in 10 min or less (McKinnon et al. 2018). This leaves patients little time for clinical decision-making and may undermine both the ‘informed’ and ‘voluntary’ dimensions of legally valid consent. Indeed, these shortcomings may not only compromise the validity of patients’ informed consent, but also risk undermining public trust in the healthcare system and the legal protection of clinicians (Gogos et al. 2011).

Importantly, existing empirical research examining ‘the folk concept’ of valid consent demonstrates that public perceptions of what constitutes sufficient information for valid consent sometimes diverge from legal or ethical standards, with laypeople often emphasising whether the person has the capacity to make autonomous decisions, rather than whether they actually exercise this capacity in a rational or well-informed manner (Demaree-Cotton and Sommers 2022). To ensure that ethical and legal standards are met and that patients are appropriately informed of their rights in relation to medical consent, it is necessary to identify such potential divergences between lay judgments and official definitions (Earp et al. 2021). However, public perceptions of consent validity—and the factors potentially affecting such perceptions—have not yet been studied in relation to AI-supported consent systems such as Consent-GPT. To begin to address this need, we examine how the deployment of different consent-seeking agents—human versus AI—influence the perceived validity of the consent obtained by such a process. This approach allows us to begin to understand potential barriers to public understanding or acceptance that could be relevant to the successful implementation of AI systems in surgical consent.

## 2 Methods

This study was designed to assess how the UK public views the validity of medical consent when key aspects of the consent-seeking process (e.g., information disclosure, answering questions, and documentation) are undertaken by a different agent: either an AI system designed to aid in the consent process (“Consent-GPT”), a junior doctor, or the treating surgeon.

Prior to conducting the main study, we conducted a pilot study (*n* = 28) to validate our experimental design and measurements. The pilot confirmed the feasibility of our approach and helped refine our measures (see Supplementary Information for details of the pilot results and study pre-registration). This study was approved and registered with the Human Research Ethics Committee at Monash University (on 21/06/2023, Project 39,035) and the Central University Research Ethics Committee at Oxford University (on 15/06/2023, R80692/RE010) (see Supplementary Information).

### 2.1 Open science

The hypotheses, sampling and confirmatory analysis plan, exclusion criteria, measures, statistical power calculations, and exploratory analyses were pre-registered at AsPredicted.org (https://aspredicted.org/ctxb-hr5d.pdf). The complete materials, anonymised data, and code to reproduce statistical analysis are available on the Open Science Framework (OSF) (https://osf.io/h3qjd/).

### 2.2 Participants

Using the *pwr* package in R, we established a target sample size of 375 (125 participants per condition) to reliably detect a small-to-medium effect (Cohen’s *f* = 0.20) with 90% statistical power, an alpha level of 0.05 and 3 numerator degrees of freedom. Anticipating exclusions, we aimed to oversample by 10%, targeting 413 participants total. We ultimately recruited 416 participants from a UK sample that was demographically representative for age, race/ethnicity, and gender on 23 November 2024. Participants were paid £1.20 for completing the short web-based survey (according to the minimum hourly wage in the UK).

We excluded data from 40 participants (exclusion rate = 9.6%) who failed an attention check (*n* = 27), or comprehension check (*n* = 14), or did not complete the survey in the pre-registered timeframe (*n* = 0), resulting in a final sample of 376 participants (52.1% self-identified as women, 46.8% as men, and 1% as non-binary or did not disclose; median age = 32 years, range from 19 to 60 years) (Table 1). The majority (*n* = 232; 61.7%) reported having previously given consent for surgery themselves. The median survey completion time was 8 min.

### 2.3 Design

**Fig. 1:**
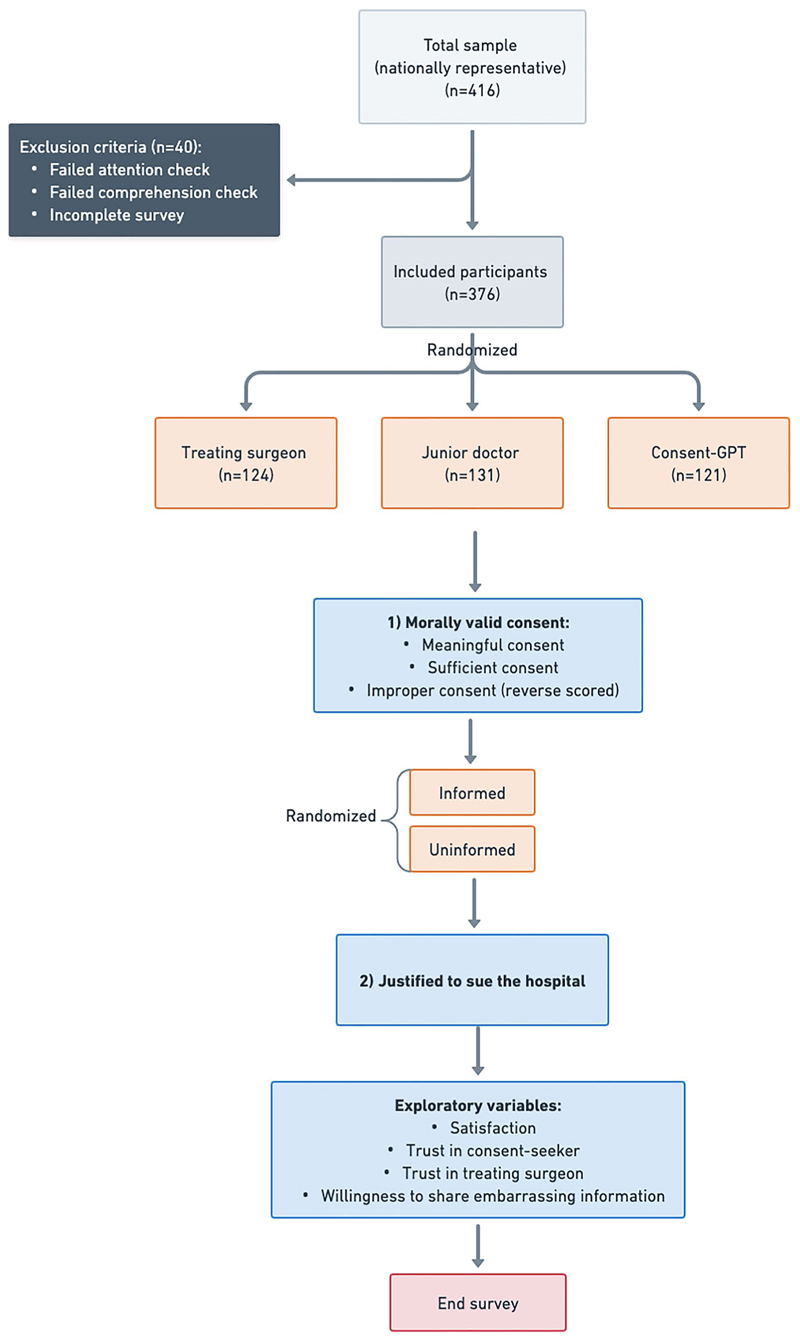
Outline of survey design. Flow diagram showing random assignment of participants to three conditions (*Consent-GPT*, junior doctor, treating surgeon). Participants viewed identical surgical consent conversation content, with only the identity of the consent-seeking agent differing across conditions. Participants were then asked to rate the validity of the consent process and how justified it would be to sue the hospital in different scenarios

Using the contrastive vignette technique to isolate the effect of consent-seeking agent on perceived consent validity and other dependent variables of interest (described below), participants were randomly assigned to one of three experimental conditions: *Consent-GPT* (*n* = 121; 32.2%), junior doctor (*n* = 131; 34.8%), or treating surgeon (*n* = 124; 33.0%) (Fig. 1). In all conditions, participants read the exact same consent conversation transcript, with identical information about risks, benefits, and alternative treatments (see **OSF repository** for the full survey materials). Additionally, all conditions specified that the treating surgeon reviewed and approved the consent documentation before the procedure. The only difference was the framing of which agent (*Consent-GPT*, junior doctor, or treating surgeon) conducted the conversation, with slight modifications to maintain ecological validity and reflect the current limitations of in-person consent practices (e.g., 10-min in-person conversations for human conditions and 2-weeks online access for *Consent-GPT* condition). All participants were then shown two hypothetical extensions to the original case scenario in random order: one in which a complication occurred that the patient (Robin) had been informed about prior to the surgery, and the other in which a complication occurred about which the patient had *not* been informed.

### 2.4 Study conditions and hypotheses

As shown in Fig. 1 and described previously, the study involved three conditions: (1) a treating surgeon condition, in which the surgeon performing the procedure obtained consent directly; (2) a junior doctor condition, in which consent-seeking was largely delegated to a junior doctor on the treating team; and (3) a *Consent-GPT* condition, in which consent-seeking was largely delegated to an AI system designed for medical consent. The treating surgeon condition served as an ecological control, representing the preferred approach within current practice constraints, while allowing us to compare both forms of consent delegation (i.e., to junior doctors and AI) against this benchmark. Based on previous research into the so-called “algorithmic bias,” which refers to relatively negative judgments toward AI-generated outcomes even when they are otherwise identical to those produced by humans (for a recent discussion and related findings, see Khan et al. 2025), we developed two pre-registered primary hypotheses:

**H1:** Perceived consent validity in the junior doctor condition would be higher than in the *Consent-GPT* condition, even when the information provided is identical.
**H2:** Participants would consider it more justifiable to sue the hospital when consent was obtained through *Consent-GPT* rather than human clinicians, given documented differences in how people attribute responsibility and liability between human and AI systems in medical decision-making contexts.

### 2.5 Procedure and materials

Each participant was presented with a vignette describing a patient (Robin) who requires a laparoscopic cholecystectomy (a minimally invasive surgical technique used to remove a diseased gallbladder). Each version of the vignette described the consent process, including how information about the procedure was communicated, how questions were answered, and how formal consent was documented. After reading about how the consent process would be conducted (i.e., with the treating surgeon, junior doctor, or *Consent-GPT*), all participants were shown the same transcript of the consent conversation (generated using GPT-4 with the prompts described below), but were told it represented the conversation between Robin and the assigned consent-seeking agent (treating surgeon, junior doctor, or *Consent-GPT*). The only systematic variation between conditions were those necessary to maintain ecological validity in how different agents would realistically engage in consent processes (e.g., 10-min in-person conversations for human conditions and 2-weeks online access for *Consent-GPT* condition). The specific vignettes by condition are shown in Table 2A.

**Fig. 2:**
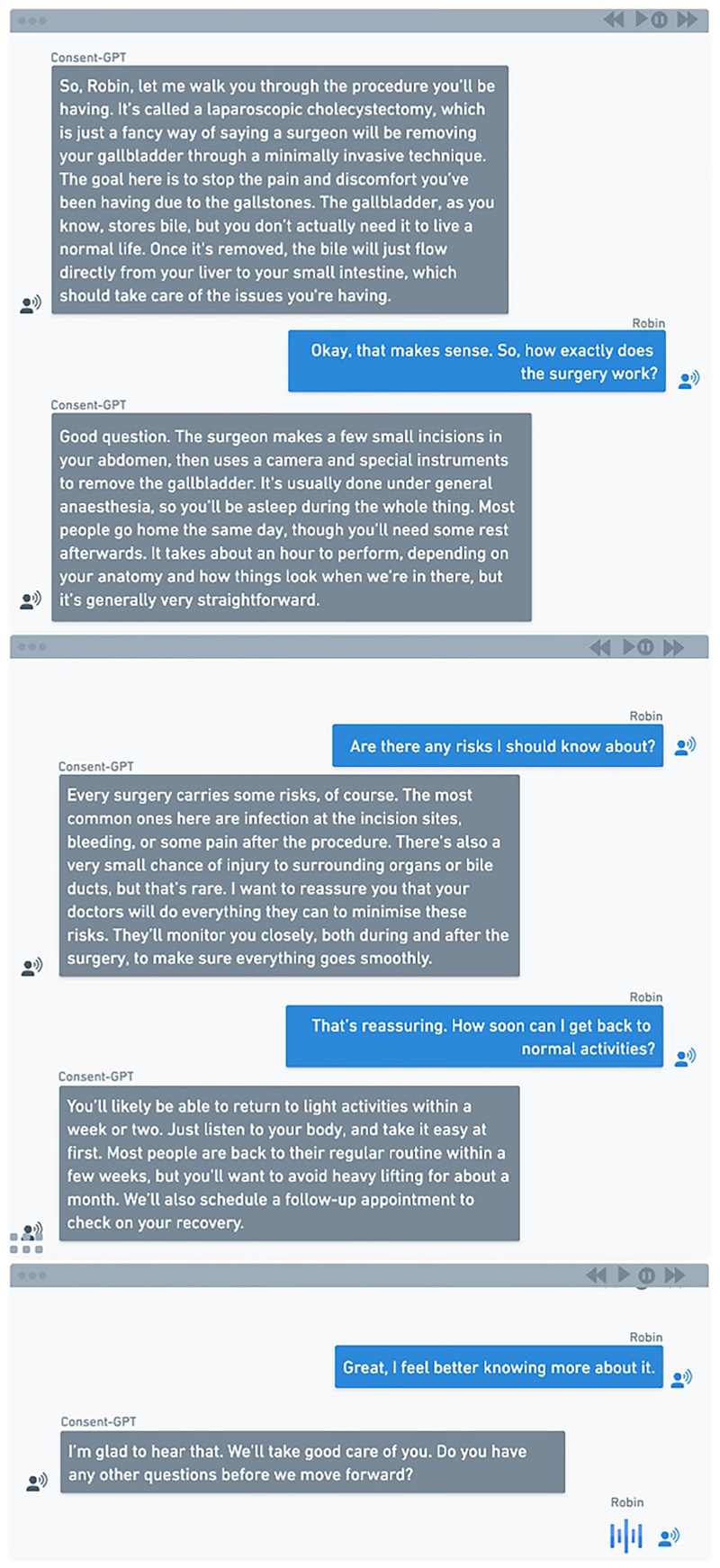
Consent conversation transcript—Consent-GPT example. Sample transcript showing dialogue between AI system and patient “Robin” regarding laparoscopic cholecystectomy, including risks, benefits, and alternatives

All participants were then shown the same consent conversation transcript with different labels for the consent agent (i.e. treating surgeon, junior doctor, or *Consent-GPT*) (Fig. 2), with the only difference being who was described as having had the conversation with Robin—the treating surgeon, junior doctor, or *Consent-GPT*.

The transcript was generated using Open-AI’s GPT-4 in response to the following prompt: “*Speak to me as if you are my surgeon providing me with the information I need to give consent for surgery. The surgery I am expecting to have is a laparoscopic cholecystectomy to treat my acute cholecystitis. I am otherwise healthy with no past medical history and no medications. Speak to me in the form of dialogue, you will be the surgeon and I will be the patient.”*

Following these transcripts, the vignettes continued with Robin indicating they had no further questions and proceeding to sign the consent form (Table 2B).

After reading the vignette, participants rated their agreement with various statements on 0–100 scales (labelled 0 = strongly disagree, 25 = somewhat disagree, 50 = neutral, 75 = somewhat agree, 100 = strongly agree).

As pre-registered, to test our first hypothesis about differences in perceived consent validity between AI and human clinicians (H1), we created a composite measure, “*perceived consent validity*” by taking the mean of three items: whether the consent was meaningful (*“Robin has given meaningful consent for the procedure to go ahead.”*), sufficient (*“The consent process was sufficient to allow the treating surgeon to go ahead with the surgery.”*), and proper (*“If the treating surgeon proceeds with the surgery now, they’ll be acting without Robin’s proper permission”*—reverse-scored). Cronbach’s α for the three-item measure was 0.87 (95% CI [0.85, 0.89], standardized *α* = 0.88), which we deemed acceptable. Item-total correlations were high for all three items (meaningful consent: *r* = 0.84; sufficient consent: *r* = 0.80; proper consent: *r* = 0.65), with an average inter-item correlation of *r* = 0.71. This measure was designed to capture public perceptions of consent validity, rather than formal legal determinations of informed consent. As Demaree-Cotton and Sommers (2022) showed, folk concepts of valid consent may differ from formal legal criteria, with public judgements tracking broader notions of ethical legitimacy rather than strict legal compliance. Our measure thus draws on key features of ethical consent being meaningful, sufficient, and properly obtained (Beauchamp and Childress 2019).

To assess our second hypothesis regarding ***justification for litigation*** (H2), participants were presented with the following text: “*Suppose a complication accidentally occurs during the procedure. This complication is a known risk of the procedure and occurred even though it was carried out carefully*.” Participants were then shown two scenarios in random order: Informed scenario (“*Before the procedure took place, [agent]* ***did mention*** *this possible complication*.”), and Uninformed scenario (“*Before the procedure took place, [agent]* ***did NOT mention*** *this possible complication*.”). For each scenario, participants rated their agreement on a scale from 0 (completely disagree) to 100 (completely agree) with the statement “*Under these circumstances, Robin is justified in suing the hospital*”.

The study included several additional exploratory measures that asked participants to imagine themselves in Robin’s position. Using 0–100 scales, participants indicated their level of agreement with the following statements: *“If I were Robin, I would feel satisfied with having gone through the consent process with [agent], based on the conversation described.”*; *“If I were Robin, I would trust [agent] to give me all the relevant information I need to consent to the procedure.”*; *“If I were Robin, the fact that consent was delegated to [agent] makes me doubt whether the treating surgeon is acting in my best interest.”*; and (for junior doctor and *Consent-GPT* conditions only), *“If I were Robin, I would feel comfortable sharing potentially embarrassing personal health information with [agent]”*.

The sliding scales were labelled with ‘strongly disagree’, ‘somewhat disagree’, ‘neutral’, ‘somewhat agree’, and ‘strongly agree’ at positions corresponding to 0, 25, 50, 75, and 100 respectively, though numerical values were not shown to participants. For analysis, responses were discretised into five ordered groups: strongly agree (77.5–100), somewhat agree (55–77.5), neutral (45–55), somewhat disagree (22.5–45), and strongly disagree (0–22.5) (unless otherwise specified in the **Results**).

The selection of exploratory measures was guided by prior research identifying key dimensions of patient-provider interactions that could influence consent processes. Patient satisfaction was included as a crucial indicator of consent quality, with research showing that satisfaction with the consent process correlates with patient understanding and engagement (Convie et al. 2020). Trust measures were incorporated given their fundamental role in obtaining valid consent in the context of therapeutic relationships (Ludewigs et al. 2022). Finally, the measure regarding comfort sharing potentially embarrassing personal health information was included based on research by Frick et al. (2021), which found that patients’ willingness to disclose sensitive medical information to conversational agents may differ from disclosure to human physicians.

Participants also rated their trust in different sources of medical information on 0–10 scales (medically specialized AI vs doctors). Finally, participants completed demographic questions about age, gender, and prior surgery experience.

### 2.6 Qualitative data collection

Participants were invited to provide optional qualitative explanations for their responses through free-text short answer boxes. Specifically, participants were prompted with “*Please briefly explain your response to the above questions on this page*” after completing the consent validity ratings and again after the litigation scenario questions.

### 2.7 Analysis

Before combining our *perceived consent validity* items into a composite score, we conducted reliability analysis using Cronbach’s α to assess their internal consistency, as specified in our pre-registration. As pre-registered, we then conducted two primary analyses of the UK sample (final *n* = 376): a one-way ANOVA examining the effect of consent-seeking agent (i.e., treating surgeon, junior doctor, or *Consent-GPT*) on perceived consent validity, and a 3 (consent agent) × 2 (informed versus not informed) mixed ANOVA examining justification to sue the hospital following a complication. The second analysis compared responses across the three consent agent conditions (between-subjects factor), while also testing how participants’ willingness to sue varied based on whether Robin had been informed or not about the complication during the consent process (within-subjects factor).

Given that our primary hypothesis (H1) concerned *perceived consent validity*, we conducted detailed distributional checks for this key outcome measure. When the consent validity composite did not meet normality assumptions, we supplemented our parametric analyses with non-parametric approaches (i.e. Kolmogorov–Smirnov test for normality, see Supplementary Table S2).

We also conducted several pre-registered exploratory analyses. These included correlation analyses between perceived consent validity and other measures such as satisfaction, trust, and comfort sharing personal health information. For the exploratory analyses, we proceeded with parametric approaches as pre-registered.

## 3 Results

### 3.1 Perceived validity of delegated consent (Hypothesis 1)

Perceived consent validity was significantly lower in the *Consent-GPT* condition compared to both the junior doctor and treating surgeon conditions (*F*(2, 373) = 19.83, *p* < 0.001). The valid consent composite measure did not follow a normal distribution in any of the three conditions (see Supplementary Information for results from Kolmogorov–Smirnov test for normality); therefore, we conducted additional non-parametric analyses following established guidelines for handling non-normal data in behavioural research (Field 2013; Wilcox 2011).

We conducted both bootstrap analyses and non-parametric tests to address the non-normal distribution. The asymptotic general independence test, a non-parametric analysis, confirmed significant differences between conditions (χ^2^ = 36.041, df = 2, p < 0.001). Bootstrap analysis with 95% confidence interval confirmed these differences: perceived consent validity in *Consent-GPT* (95% CI [82.33, 89.67]) was rated significantly lower than in both junior doctor (95% CI [93.33, 98.33]) and treating surgeon (95% CI [97,100]) conditions, with no overlap between confidence intervals. Therefore, non-parametric analyses showed no significant differences between the junior doctor and treating surgeon conditions, while both were rated significantly higher than the *Consent-GPT* condition.

**Fig. 3:**
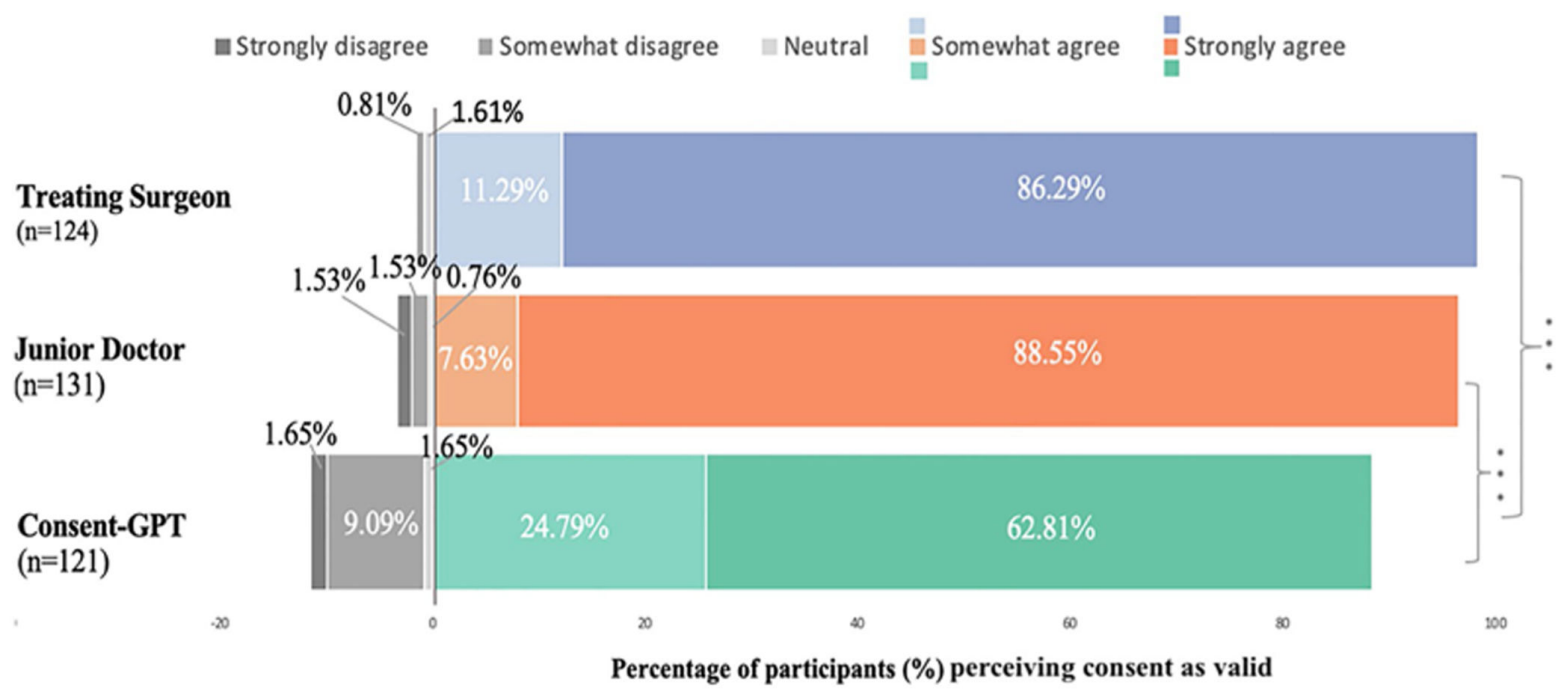
Frequency of responses to perceived validity of the consent process by condition (n = 376). Distribution of participant ratings across treating surgeon (blue), junior doctor (orange), and *Consent-GPT* (green) conditions. Stacked bars represent the percentage of participants in each response category: strongly agree (77.5–100), somewhat agree (55–77.5), neutral (45–55), somewhat disagree (22.5–45), and strongly disagree (0–22.5). *p < 0.05, **p < 0.01, ***p < 0.001

However, follow-up descriptive analyses revealed that perceived validity was generally high across all conditions (Fig. 3). Responses were measured using a 100-point sliding scale (see 2.5 Procedure and Materials for details on how responses were categorized). Using these categories, in the treating surgeon condition, 86.29% of participants strongly agreed that they provided valid consent and an additional 11.29% somewhat agreed, with only 2.42% rating neutral or below the midpoint. Similarly, for the junior doctor, 88.55% strongly agreed and 7.63% somewhat agreed, with only 3.82% rating neutral or below the midpoint. *Consent-GPT* still received predominantly positive ratings, with 62.81% strongly agreeing and 24.79% somewhat agreeing that valid consent had been obtained, though 12.39% rated it neutral or below the midpoint of the scale.

Participants’ qualitative responses provided additional insight into these ratings.^5^ Some recognized potential benefits of AI-assisted consent, with one participant noting that “the AI probably explained it more clearly than a real doctor would.” Others expressed concerns about the absence of human interaction, stating that “the consent feels less meaningful than giving consent to a real person.” For the human conditions, participants emphasized the value of direct communication, as illustrated by one response about the treating surgeon condition: “The actual surgeon explained everything and Robin agreed to it. Very clear”.

### 3.2 Justified to sue (Hypothesis 2)

**Fig. 4:**
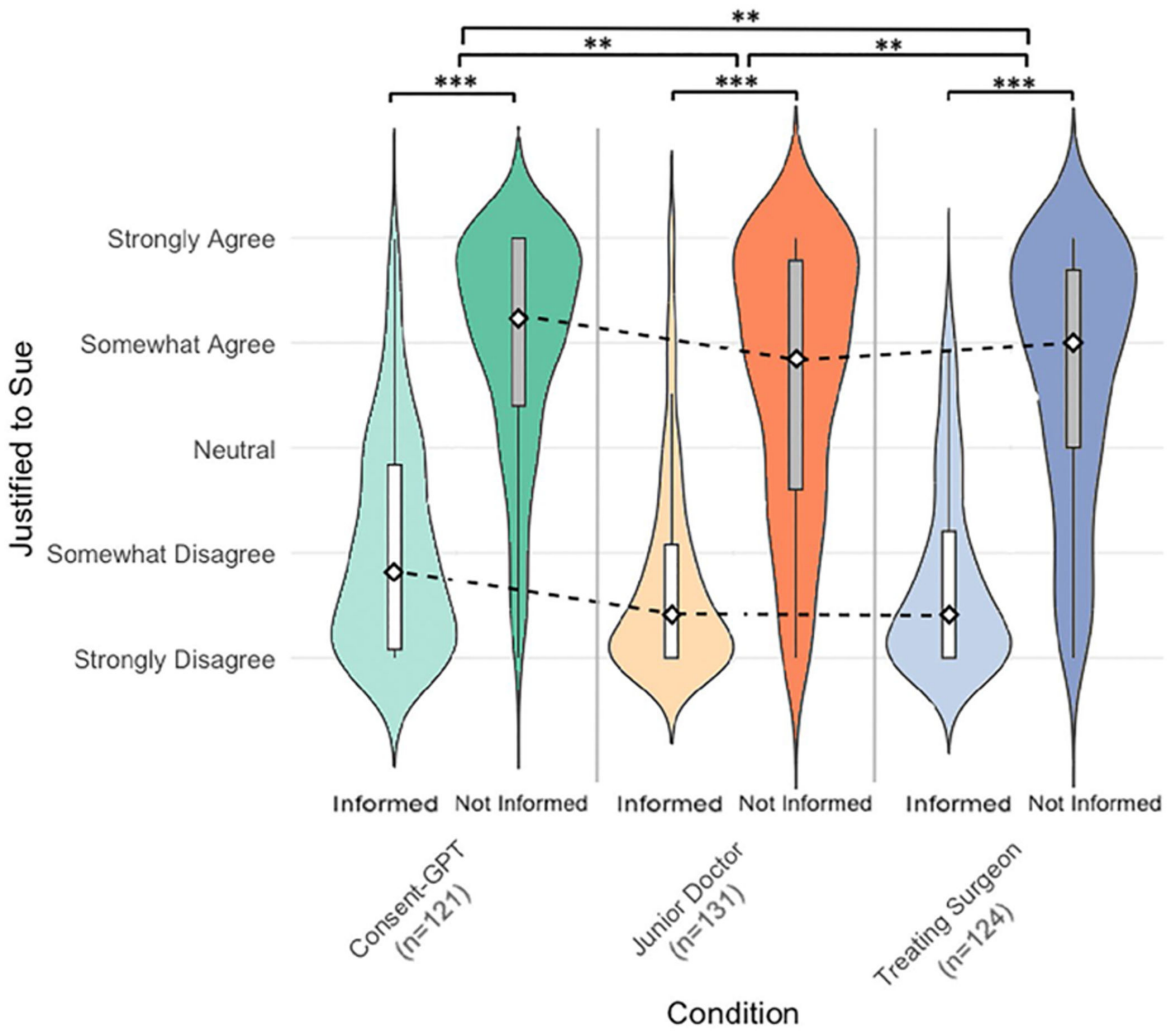
Violin plots of participants’ responses to “justified to sue” based on informed status (*n* = 376). Distribution of judgments about justification to sue across three consent agent conditions (treating surgeon: blue; junior doctor: orange; *Consent-GPT*: green). Darker shade violin plot and dark grey boxplot: complications disclosed during consent; lighter shade violin plot and white box-plot: complications not disclosed. Width indicates response density on 0–100 scale, where 0 = “strongly disagree”, 25 = “somewhat disagree”, 50 = “neutral”, 75 = “somewhat agree”, and 100 = “strongly agree”. *p < 0.05, **p < 0.01, ***p < 0.001

Participants’ judgments about the justification to sue the hospital varied significantly based on whether they were informed about complications during the consent process (Fig. 4). A 3 × 2 mixed ANOVA revealed a significant main effect of both informed status (*F*_(1, 373)_ = 873.97, *p* < 0.001) and consent agent condition (*F*_(2, 373)_ = 6.23, *p* = 0.002).

When complications had been disclosed during consent (informed condition), support for legal action was consistently low across all consent agents (*Consent-GPT Mdn* [median] = 20, *IQR* [interquartile range] = 44; junior doctor *Mdn* = 10, *IQR* = 27; treating surgeon *Mdn* = 10, *IQR* = 30). However, when complications had not been disclosed (Uninformed condition), support for suing increased substantially across all consent agents (*Consent-GPT Mdn* = 81, *IQR* = 40; junior doctor *Mdn* = 71, *IQR* = 54.5; treating surgeon *Mdn* = 75, *IQR* = 42.25).

While participants were somewhat more inclined to support legal action when consent was obtained by *Consent-GPT* compared to human clinicians, the lack of significant interaction between agent and informed status (*F*(2, 373) = 0.05, *p* = 0.955) indicates that the impact of non-disclosure on willingness to sue was similar regardless of which consent agent failed to mention the complication.

Interestingly, an informal look at participants’ comments reflected varying perspectives on liability standards across the different consent conditions. In the *Consent-GPT* condition, participants often emphasized institutional responsibility, as illustrated by one response: “If the AI did not tell Robin, then that’s withholding medical information so Robin was unable to make a fully informed decision (despite thinking they did) and the hospital are at fault for using this technique.” In contrast, comments in the treating surgeon condition often focused on the inherent limitations of risk disclosure, with one participant noting “The surgeon cannot reasonably be expected to describe every possible risk or complication.” The junior doctor condition elicited mixed responses about liability, with some participants expressing confidence in the capability of junior doctors (“Junior doctors are qualified to get consents… knew the procedure, knew the risks”) while others showed hesitation (“Unfortunately it was a junior DR who gave the info and not the surgeon”). Some participants, regardless of condition, emphasized patient responsibility in the consent process, with comments like “everyone should have a basic understanding before consent that things can go wrong.” A few participants also raised broader healthcare system concerns, as exemplified by one response: “Why on earth would you sue a hospital? You’re just taking away money for them to treat other people.”

### 3.3 Exploratory findings

**Fig. 5:**
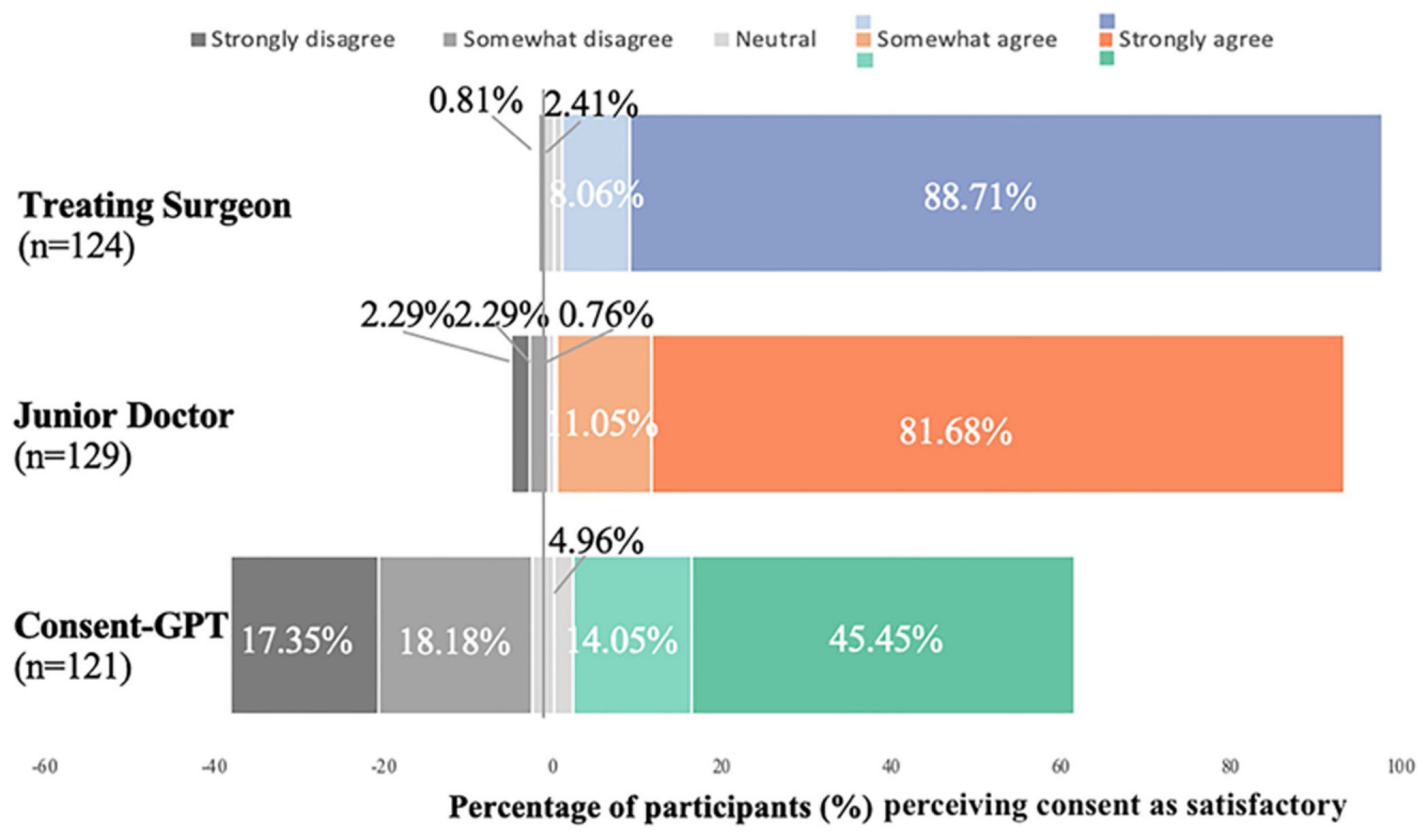
Frequency of responses to whether the consent process was considered satisfactory, by condition (*n* = 374). Distribution of satisfaction ratings across treating surgeon (blue), junior doctor (orange), and *Consent-GPT* (green) conditions. Bars represent percentage of participants selecting each response category on 100-point scale, where 0 = “strongly disagree”, 25 = “somewhat disagree”, 50 = “neutral”, 75 = “somewhat agree”, and 100 = “strongly agree”. Responses were categorized as: strongly agree (77.5–100), somewhat agree (55– 77.5), neutral (45–55), somewhat disagree (22.5–45), and strongly disagree (0–22.5)

*Satisfaction with the consent process* Analysis of participants’ satisfaction with the consent process revealed striking differences between conditions (Fig. 5). The treating surgeon condition received the highest satisfaction rating, with 88.71% of participants strongly agreeing and an additional 8.06% somewhat agreeing that the process was satisfactory. Only 0.81% somewhat disagreed, and notably, no participants strongly disagreed. Similarly, the junior doctor condition received high satisfaction ratings, with 81.68% strongly agreeing and 11.45% somewhat agreeing, while only 2.30% strongly disagreed.^6^

In contrast, the *Consent-GPT* condition showed greater variance in satisfaction levels: while 45.45% strongly agreed and 14.05% somewhat agreed that the process was satisfactory, there were also substantial proportions who disagreed (18.18% somewhat disagreed and 17.36% strongly disagreed). Qualitative responses suggested this variance might relate to concerns about human interaction, with one participant in the *Consent-GPT* condition noting “I do feel that if there had been a live doctor in person, Robin might have read body cues and signs to enable him to ask more questions.” Some participants proposed potential solutions, suggesting a “hybrid approach” where AI could be “integrated but shouldn’t become the only form of consent.”

**Fig. 6:**
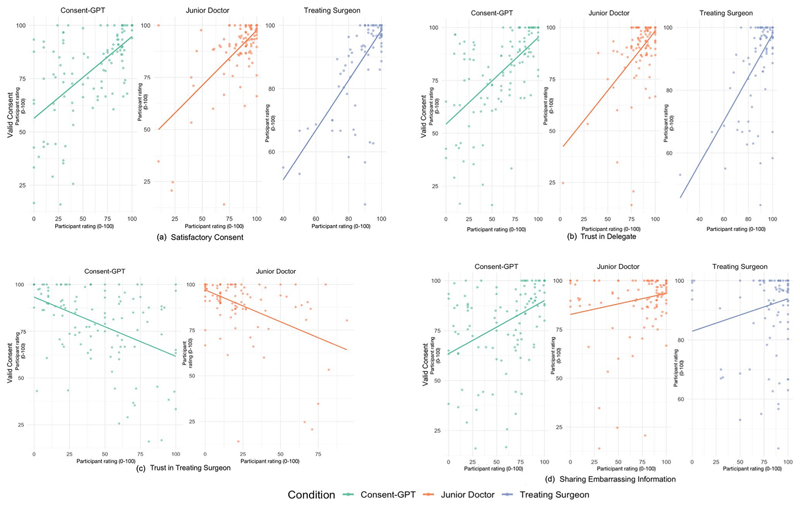
Pearson correlation between perceived validity of consent and related measures (*n* = 376). Scatterplots showing correlations between perceived validity of consent (x-axis) and four key variables (y-axis) across experimental conditions (treating surgeon: blue dots; junior doctor: orange dots; *Consent-GPT*: green dots). Panel (from left to right) (**a**) shows correlation with participant satisfaction with the consent process (*r* = 0.613–0.735); panel (**b**) shows correlation with trust that the agent provided all relevant information (*r* = 0.577-0.629); panel (**c**) shows correlation with doubt about the treating surgeon’s decision to delegate consent (*r* = – 0.463 to – 0.487, reverse-scored); and panel (**d**) shows correlation with comfort sharing potentially embarrassing personal health information (*r* = 0.203–0.422). Axes represent 0–100 scale, where 0 = “strongly disagree”, 25 = “somewhat disagree”, 50 = “neutral”, 75 = “somewhat agree”, and 100 = “strongly agree”. No results for panel (**c**) in treating surgeon condition as this question was not applicable of consent process and trust that the consent agent will provide information relevant for consent (treating surgeon (*r* = 0.629, *p* < 0.001), junior doctor (*r* = 0.577, *p* < 0.001), and *Consent-GPT* (*r* = 0.608, *p* < 0.001)) (Fig. 6B).

As an exploratory analysis, we examined the relationship between satisfaction and consent validity (Fig. 6A). Analyses revealed positive correlations with perceived consent validity across all conditions, with the strongest relationship observed in the treating surgeon condition (*r* = 0.735, *p* < 0.001), followed by the junior doctor (*r* = 0.644, *p* < 0.001) and *Consent-GPT* (*r* = 0.613, *p* < 0.001) conditions.

*Trust in the Consent Process* Across all conditions, we found a positive correlation between perceived validity of consent process and trust that the consent agent will provide information relevant for consent (treating surgeon (*r* = 0.629, *p* < 0.001), junior doctor (*r* = 0.577, *p* < 0.001), and *Consent-GPT* (*r* = 0.608, *p* < 0.001)) (Fig. 6B).

Additionally, trust in the treating surgeon’s decision to delegate consent showed moderate negative correlations with consent validity ratings in both *Consent-GPT* (*r* = – 0.463, *p* < 0.001) and junior doctor (*r* = – 0.487, *p* < 0.001) conditions (Fig. 6C), suggesting that higher perceived validity of consent was associated with lower doubt about the treating surgeon’s motives for delegation.

Comfort in sharing embarrassing personal health information showed varying correlations with consent validity across conditions. The strongest relationship was found in the *Consent-GPT* condition (*r* = 0.422, *p* < 0.001), with weaker positive correlations in the junior doctor (*r* = 0.221, *p* = 0.011) and treating surgeon (*r* = 0.203, *p* = 0.024 conditions (Fig. 6D).

*Trust in medical information sources* Participants expressed significantly higher trust in their doctors (*M* = 8.36, SD = 1.47) compared to medically specialized AI (*M* = 4.12, SD = 2.94) for accurate medical advice (*t*(375) = – 26.03, *p* < 0.001). A positive correlation (*r* = 0.456, *p* < 0.001) was found between trust in AI accuracy and perceived validity of consent in the *Consent-GPT* condition, suggesting that favourable attitudes towards AI technology were associated with perceptions of AI-supported consent.

## 4 Discussion

This study represents the first empirical investigation into how laypeople view two critical but understudied aspects of medical consent: the potential role of AI systems in consent processes and the broader practice of consent delegation in healthcare.

### 4.1 Perceptions of consent validity between AI versus human consent agents

While participants generally agreed that AI-assisted consent was valid, they indicated significantly lower levels of agreement compared to when consent was obtained solely by human clinicians. These findings align with a growing body of research on public attitudes towards AI in healthcare. Although studies examining early applications of AI in consent processes have shown promising results for patient engagement and satisfaction (Aydin et al. 2023), with some evidence that AI-supported consent may even improve patient understanding of relevant clinical information (Aydin et al. 2023; Xiao et al. 2023), research consistently shows that patients and healthcare professionals prefer human-led decision-making and explainable AI, even if it means slightly compromised accuracy (Rojahn et al. 2023; Busch et al. 2024). In particular, people tend to prefer human involvement for complex communication tasks that require trust-building (Sassi et al. 2024), especially for more complex medical decisions rather than administrative or logistical tasks (Nov et al. 2023).

The acceptance of AI tools in healthcare appears to be modulated by factors including direct AI experience, technological literacy, education level, and health status (Busch et al. 2024; Zhang et al. 2020). Research investigating AI diagnostic tools shows that while patients accept AI assistance, they prefer final decisions to come from human clinicians (Goodman et al. 2023). Additionally, as this technology becomes more prevalent in healthcare, there is a risk that clinicians may become over-reliant on AI systems, or fail to engage critically with the information provided, potentially undermining patients’ informed decision-making if the LLM provides false or misleading information. This phenomenon manifests particularly in fast-paced healthcare environments where cognitive resources are strained (Ho and Vuong 2025; Goddard et al. 2012).

The preference for human involvement is particularly interesting given recent research showing that when participants are unaware of the source, LLM-generated responses to patient questions are often rated as *more* empathetic than human physician responses (Ayers et al. 2023). This apparent contradiction might be explained by how knowledge of AI involvement affects perceptions of the interaction. An important consideration for future AI implementation is the emerging evidence that individuals can form meaningful relationships with AI agents over time, which may significantly alter these initial preference patterns (Reverberi et al. 2022; Earp et al. 2025).

The changing nature of human–AI interaction in healthcare is characterized by increasingly sophisticated co-decision-making processes that move beyond simple automation toward more “collaborative” interactions (Krügel et al. 2022; Porsdam Mann et al. 2024). Research demonstrates that effective human–AI co-operation emerges through dynamic, adaptive processes where both humans and AI systems learn from each other’s capabilities and limitations (Puranam 2021). In healthcare specifically, there is evidence that clinicians develop nuanced reliance strategies, following AI advice more when it aligns with their clinical judgment while maintaining appropriate scepticism when recommendations seem incongruent (Reverberi et al. 2022).

While there may be certain cases where expert-LLM collaborations have been shown to outperform either alone (for example, in responses to 21 common patient questions about retinal disease (Tailor et al. 2024), a meta-analysis by Vaccaro et al. (2024) found that human-AI combinations often underperformed in tasks like clinical decision-making and diagnosis. However, the evolving nature of human–AI interaction suggests that patients’ initial hesitations about AI-supported consent might diminish as they develop more sophisticated mental models of AI capabilities and limitations (Bansal et al. 2019). Moreover, some researchers argue that AI’s capacity for consistent, rational compassion might actually be advantageous in medical contexts, as it avoids the emotional burnout that can affect human healthcare providers (Inzlicht et al. 2024). However, since our study did not directly measure perceptions of empathy or compassion, further research is needed to understand how these factors might influence patients’ preferences regarding AI involvement in consent processes.

### 4.2 Perceptions of legal implications of consent delegation

Our findings on litigation attitudes reveal that while participants were more inclined to support litigation when consent was obtained through AI rather than human clinicians, the strongest predictor was whether complications had been properly disclosed during the consent process, regardless of the consent-seeking agent. This aligns with research showing that failure to properly disclose complications is the most common reason for complaints involving the consent process (Gogos et al. 2011). Indeed, negligence claims for lack of informed consent have risen fourfold in the UK since 2015, costing the NHS approximately £62 million annually (Dyer 2023; Wald and Kelly 2021). A study of nearly 10,000 malpractice claims in Australia found that 9% of consent disputes centred on whether specific risks should have been disclosed, with two-thirds involving surgical procedures (Bismark et al. 2012). Our finding that people were substantially more likely to support litigation (i.e., suing the hospital) when complications were not disclosed, regardless of whether consent was obtained by AI or human clinicians, suggests that people hold both human and artificial agents to broadly similar standards regarding risk disclosure.

Future research may wish to identify if there are significant regional differences in support for legal action, particularly comparing jurisdictions with different legal frameworks or varying cultural attitudes toward litigation in healthcare settings. For example, attitudes towards risk disclosure and acceptable delegation may vary in contexts where shared or family-based decision-making traditions are stronger (Wong and Wang 2021). This is particularly significant given that many healthcare systems are shifting towards a “particular patient” approach to disclosure (known as the *Montgomery standard* in the UK), where doctors are expected to provide specific, individualized information about risks, regardless of rarity (NHS 2024).

The slightly higher inclination to support litigation in AI-assisted consent cases appears to reflect broader issues of trust and liability in healthcare systems. Studies show that patients frequently misunderstand the fundamental purpose of informed consent, with less than half (41%) believing that the consent process helps make their preferences known to treating teams (Akkad et al. 2006). Instead, many view consent primarily as legal protection for healthcare institutions rather than as a process to enhance patient autonomy and informed decision-making (Sherlock and Brownie 2014). This misalignment is exacerbated by current documentation practices, where written consent forms are often viewed by both patients and clinicians as a “ritualistic” legal requirement, rather than a meaningful tool for patient engagement (Convie et al. 2020). Research indicates that fewer than half of patients actually read the consent forms they are given (Agozzino et al. 2019; Özhan et al. 2014), and many forms use complex language that patients struggle to understand (Paasche-Orlow et al. 2003). Some scholars argue that this emphasis on documentation can lead to “consent desensitization,” where individuals no longer make active informed choices when providing consent (Schermer et al. 2014).

### 4.3 Cultural considerations and AI bias

Our findings reflect a predominantly Western, individualistic perspective that may require contextualization within broader cultural frameworks. Cross-cultural research demonstrates significant variation in how different cultures conceptualize autonomy and consent (Wong and Wang 2021). The Japanese *Society 5.0* framework offers an alternative approach, incorporating concepts like “mín běn” (民本) that emphasize collective wellbeing alongside individual autonomy (Ho and Luu 2024). In cultures with family-based decision-making, AI systems would need to be designed to facilitate multi-party consent processes that respect traditional authority structures while protecting individual patient interests (see also our discussion of AI-assisted pediatric consent, which also involves multiple parties (Allen et al. 2025). Therefore, successful AI implementation requires careful consideration of cultural factors, relational context, and acknowledgement of varied approaches to medical decision-making.

Recommended strategies to address some of these cultural concerns may include incorporation of governance structures that engage stakeholders from all backgrounds during AI development (Ho and Luu 2024), training AI systems on diverse patient populations, and developing ongoing feedback loops with active human monitoring (Jung 2024). AI-supported consent systems should also ensure digital interfaces are adapted for different cultures, including use in multiple languages and with features that accommodate diverse literacy and technological capabilities.

### 4.4 Towards a hybrid model for consent delegation

Our findings suggest that AI implementation is more likely to improve (rather than amplify) existing challenges regarding the “ritualistic” nature of informed consent, though with important caveats about maintaining human involvement. The predominantly positive validity ratings for AI-supported consent (87.60% agreed it was valid, with 62.81% strongly agreeing) indicate that participants saw fundamental merit in the approach, even while preferring human alternatives. This finding, combined with participants’ qualitative responses recognizing AI’s potential for clearer explanations (e.g., “the AI probably explained it more clearly than a real doctor would”), suggests that AI could address documented problems in current consent practices such as time constraints, inconsistent information delivery, and junior doctors’ limited training (Wood et al. 2016; Propst et al. 2019). However, the substantially lower satisfaction ratings for AI-supported consent (with 35.54% disagreeing that the process was satisfactory, compared to only 2.30% for junior doctors) indicate that too much delegation to AI may amplify concerns about depersonalization and loss of human connection that already exist in current rushed consent practices. While there are concerns about added complexity in accountability, AI systems could potentially disrupt the status quo in consentseeking practices by offering patients more time and opportunities for detailed discussions than busy clinicians can typically provide.

Nevertheless, it remains crucial to consider that patients value the consent process as an opportunity for meaningful dialogue and recognition of their individual perspectives. Research shows that patients often ascribe greater importance to “feeling” informed and understood, rather than simply receiving specific information about procedures (Convie et al. 2020). A qualitative meta-aggregation of patient experiences with surgical consent found that the interpersonal aspects of consent discussions (i.e., feeling heard, respected, and individually valued) were central to patient satisfaction with the process (Convie et al. 2020).

It is also worth highlighting Wilkinson and Levy’s theoretical framework of “scaffolded autonomy,” which recognizes that autonomous decision-making requires not just information delivery, but appropriate social and epistemic support structures that help patients understand and apply their values to complex clinical choices (Wilkinson and Levy 2024). The key question then becomes whether AI systems could enhance or diminish this crucial interpersonal dimension to consent. While there are concerns about the latter, AI’s potential to provide clear, comprehensive, and unhurried discussions could improve patient engagement in the consent process.

### 4.5 Limitations

Several limitations warrant discussion. Our vignette-based methodology may not fully capture the nuances of real-world consent interactions, where non-verbal communication and rapport-building play crucial roles (Convie et al. 2020). Our experimental design, which presents identical consent conversations across conditions, represents a simplified scenario and actual implementation of AI in consent processes would need mechanisms for individualised risk assessment and discussion of treatment alternatives.

Our study also faces the problem of affective forecasting (discordance between hypothetical predictions and real medical decision-making) (Ellis et al. 2018). For this reason, we also included practical considerations (10-min in-person conversations for human conditions versus 2-weeks online access for AI) to reflect current clinical realities in consent practices. Research shows that current in-person consent discussions are often time-constrained (Wood et al. 2016; McKinnon et al. 2018). However, this approach does not represent the optimal or ethically ideal consent scenario. By contrast, extended access in the AI condition represents a realistic potential advantage of this technology.

Our focus on a single specific procedure (laparoscopic cholecystectomy) limits generalizability across different medical contexts that vary in risk level, invasiveness, and complexity. Our study examined attitudes at a single point in time. Given the novelty of generative AI in healthcare, longitudinal studies would be valuable to track how perceptions towards AI may change with increased exposure to these systems over time. Furthermore, while our composite validity score showed strong internal consistency (Cronbach’s *α* = 0.87), this measure is novel and would benefit from additional studies to confirm its validity and reliability. Our study lacks comparative data to contextualise our findings because, to our knowledge, no empirical studies have examined patient attitudes towards existing consent delegation practices and perceived litigation justifiability.

Additionally, our focus on individual patient perspectives did not fully address potential system-level benefits of AI-supported consent, such as standardization of information delivery, reduced time pressure, and the ability to give patients extended access to information (Wood et al. 2016).

Finally, we tested only one format of AI-supported consent, rather than exploring different hybrid approaches with varying degrees of human involvement. This was a deliberate choice: as the first empirical study examining public attitudes towards AI in consent processes, we aimed to establish baseline perceptions by comparing clearly differentiated conditions (i.e. fully human versus AI-supported consent). While this approach allows for clear comparisons across consent agents, it means we cannot yet draw conclusions about how different degrees or types of AI–human collaborations in the consent process might affect public perceptions. Moreover, we did not directly measure perceived empathy through validated measures. Another important area for future research will be examining how patients conceptualize AI systems in consent processes. Specifically, whether they view AI as a sophisticated tool (similar to a diagnostic algorithm) or as a member of the healthcare team with quasi-social characteristics.

## 5 Conclusion

Future research priorities include examining hybrid approaches that combine AI efficiency with human involvement, investigating healthcare professionals’ perspectives on AI-supported consent delegation, and conducting longitudinal studies to track evolving public attitudes as AI becomes more prevalent in healthcare settings (see Box 2 for comprehensive recommendations).

Healthcare systems should balance potential efficiency gains against the need to maintain public trust and patient satisfaction in the consent process. Given that identical information during the consent process still resulted in lower perceived validity for AI-supported consent, implementation strategies should prioritize hybrid models that leverage AI’s systematic capabilities, while preserving the human elements that patients value in medical consent processes.

## Supplementary Material

Syupplementary Material

## Figures and Tables

**Table 1:** Participant data characteristics from the final sample following exclusions.

Characteristics	Frequency (percentage)
Total sample	
Included	376 (90.4%)
Excluded	40 (9.6%)
Gender	
Woman	196 (52.1%)
Man	176 (46.8%)
Non-binary	2 (0.5%)
Self-described	0 (0.0%)
Prefer not to say	2 (0.5%)
Prior surgery experience	
Yes	232 (61.7%)
No	121 (32.2%)
Don’t remember	4 (5.1%)
Prefer not to say	19 (1.1%)
Age (median)	32 years

Demographic characteristics of study participants (*N* = 376)

Participants whose data were excluded (*n* = 40) represented 9.62% of the original sample

**Table 2:** Vignettes by experimental condition

(A)^i^			
Intro paragraph (all conditions):			
*Robin has a medical condition requiring a surgical procedure to treat it. The surgery is expected to benefit Robin, but it also carries some risks.* * Robin wishes to have the procedure, but wants to know more about it before it goes ahead. Because treating surgeons tend to be very busy, some of them choose to ask another member of the treating team to explain the details of the procedure (including the potential risks and benefits) and ask for the patient’s consent*			
Treating surgeon condition	Junior doctor condition		*Consent-GPT* condition
*However, Robin’s surgeon, who will be performing the procedure on the day, decides NOT to delegate this part of the consent process* *Instead, the surgeon personally explains the procedure to Robin. The conversation with the surgeon lasts **10 min** and takes place **in-person** at the hospital **on the morning of the procedure*** *Afterwards, a written note of their conversation is made in Robin’s medical records*	*Robin’s surgeon decides to delegate this part of the consent process to a junior doctor who is part of Robin’s treating team, but who will NOT be performing the procedure on the day* *The conversation with the junior doctor lasts* ***10 min*** *and takes place **in-person** at the **hospital on the morning of the procedure*** *Afterwards, a written note of their conversation is made in Robin’s medical records **so** that the treating surgeon knows Robin’s preferences and concerns before the procedure*		*Robin’s surgeon decides to delegate this part of the consent process to a new AI-powered app, called Consent-GPT. The app has been approved for use in medical consent (but will NOT be involved in performing the procedure on the day)* *The conversation with the app takes place **online** and Robin can **access the app at any time in the 2 weeks before the procedure*** *Consent-GPT also includes a digital transcript of their conversation in Robin’s medical records so that the treating surgeon knows Robin’s preferences and concerns before the procedure*
(B)^ii^			
Treating surgeon condition	Junior doctor condition		*Consent-GPT* condition
*After Robin’s other questions and concerns had been addressed, Robin then **signed the consent form**, indicating their willingness to go ahead with the procedure*	*After Robin’s other questions and concerns had been addressed, Robin then **signed the consent form**, indicating their willingness to go ahead with the procedure* *This was checked over by the treating surgeon before the procedure*	*After answering a few more questions and addressing Robin’s concerns, Robin then **signed the consent form**, indicating their willingness to go ahead with the procedure* *This was checked over by the treating surgeon before the procedure*	

Comparison of vignette text across three experimental conditions: treating surgeon, junior doctor, and Consent-GPT. The introductory paragraph was identical across all conditions, with subsequent text varied to reflect different consent-seeking agents. Text has been shown in bold or underlined to reflect how it was presented to participants

Continuation of vignette text showing how the conclusion of the consent process was described across the three experimental conditions, highlighting differences in how consent was documented and reviewed. Text has been shown in bold or underlined to reflect how it was presented to participants

## Data Availability

The complete materials, anonymised data, and code to reproduce statistical analysis are available on the Open Science Framework (OSF) (https://osf.io/h3qjd/).

## 1.1 Box 1 Delegation and responsibility: assumptions of the manuscript

For clarity, when discussing ‘delegating consent-seeking’ to AI including LLMs, we refer specifically to partially delegating certain aspects of the informed consent process, such as information provision/elaboration and documentation, rather than delegating the entire process. On many philosophical accounts, AI cannot itself meaningfully ‘take’ consent insofar as it cannot bear moral responsibility. Throughout this manuscript, we assume that human clinicians remain integral to the process, including validating and bearing responsibility for consent obtained with AI assistance. The clinician would also remain responsible for several core elements of obtaining valid consent, including assessment of the patient’s decision-making capacity, evaluation of whether the patient’s decision is made voluntarily, and ensuring that the information communicated meets legal standards for informed consent.

## Declarations

1 The term “junior doctor” is somewhat controversial, with recommended terminology recently shifting towards “resident doctors.” In the UK, this includes Foundation Year doctors (FY1-FY2) and specialty trainees at various levels (Core Training, Specialty Training ST1-ST8) who have not yet reached Consultant level. The distinction between surgical registrars and surgical residents is important, as more senior trainees (e.g., ST6-ST8) would typically be more appropriate for obtaining consent for complex surgical procedures than foundation year doctors.

2 While the legal requirements for individualised consent (e.g., under *Montgomery* in the UK) raise important questions about AI implementation, the focus of this study is on examining laypeople’s views about the ethical validity of AI-supported consent rather than providing a legal analysis of whether such systems would meet current legal standards for valid consent.

3 Indeed, not only the public, but also attitudes of doctors themselves should be studied in future work. For example, if too much of the consent process is delegated to AI, junior doctors might feel they have fewer opportunities to practice and develop crucial communication skills. On the other hand, LLM-supported consent could also help to address educational gaps. For example, LLMs might enhance learning and communication skills by providing consistent models of effective consent practices, or allow physicians more time to focus on nuanced interpersonal aspects of patient care.

4 This methodology builds on a growing body of research demonstrating that people evaluate identical outputs differently based on whether they believe they were produced by AI or humans [e.g., Khan et al. (2025)]. This bias against AI-generated content has been documented even for collaborative works between humans and AI, though it can be partially mitigated when framed as augmenting rather replacing human input (Earp et al. 2024) Recent work on the “credit/blame asymmetry” of AI outputs suggests that while people readily blame AI/human collaborations for harmful outcomes (requiring only recklessness or negligence), they may be more reluctant to give credit for beneficial outcomes unless there is substantial human involvement (Porsdam Mann et al. 2023).

5 While participant quotes are included throughout this section to illustrate quantitative findings, these are selected examples only and no formal qualitative analysis was conducted. The quotes should be interpreted as illustrative examples rather than systematic qualitative findings.

6 While the full correlation matrix examining relationships between all measured variables can be found in the Supplementary Information, these specific correlations were highlighted due to their theoretical relevance to understanding how different consent delegates might impact the perceived validity of the consent process.
