# Supplementary material for: Is Consent-GPT valid? Public attitudes to generative AI use in surgical consent
Source: AI Soc. 2025 Oct 9;41(3):2637–55. doi: 10.1007/s00146-025-02644-9 (PMC7618318; doi:10.1007/s00146-025-02644-9)
Supplement: Supplementary file 1 — Supplementary file1 (DOCX 31 KB) [file 146_2025_2644_MOESM1_ESM.docx]

**Supplementary Information for**

***Is Consent-GPT valid? Public attitudes to generative AI use in surgical consent***

*Jemima W. Allen, Ivar R. Hannikainen, Julian Savulescu, Dominic Wilkinson, and Brian D. Earp*

**Supplementary Note 1**.

*Pilot Data*

We conducted a small pilot of 31 participants from the UK general public on Wednesday 20th November 2024. The sample was balanced for gender with 15 participants identifying as women (53.57%) and 13 as men (46.43%), with no participants identifying as non-binary or self-described. A total of 28 participants were included in the study (n=3 were excluded: 2 failed the attention check [6.45%], 1 failed the comprehension check [3.23%], and 0 failed to complete the survey). Participants were distributed across conditions with 8 in the treating surgeon condition, 9 in the junior doctor condition, and 11 in the Consent-GPT condition. The median age of the sample was 37.5-years-old. Of the participants, 16 (57.14%) had previously consented to surgery themselves, 11 (39.29%) had not, and 1 (3.57%) could not remember. The median survey completion time was 488 seconds (8 minutes 7 seconds).

**Supplementary Note 2.**

*Open Science and* *Power Analysis*

The hypotheses, sampling and analysis plan, exclusion criteria, measures, statistical power calculations, and exploratory analyses were pre-registered at AsPredicted.org (# 187,429).

Using the *pwr* package in *R*, we established a target sample size of 375 (125 participants per condition) to reliably detect a small effect (Cohen’s f=0.20) with 90% statistical power and an alpha level of 0.05 and 3 numerator degrees of freedom. Anticipating exclusions, we aimed to recruit an additional 10%, targeting 413 participants total.

**Supplementary Table S1.**

*Median Scores and Interquartile Ranges for Three Measures of Consent Validity by Condition.*

|  | **Meaningful consent,**  **median (IQR)** | **Sufficient consent,**  **median (IQR)** | **Proper consent,**  **Median, *reverse-scored* (IQR)** |
| --- | --- | --- | --- |
| Consent-GPT | 88 (29) | 81 (30) | 92 (25) |
| Junior doctor | 98 (11) | 95 (12) | 100 (10) |
| Treating surgeon | 100 (10) | 100 (10) | 100 (2.25) |

*Footnote: Participants were asked to respond using a 0-100 sliding scale with the following labels: 0 (strongly disagree), 25 (somewhat disagree), 50 (neutral), 75 (somewhat agree), 100 (strongly agree).*

**Supplementary Table S2.**

*Kolmogorov-Smirnov (KS) Test Results for Normality of Valid Consent Composite Measure Distribution Across Conditions.*

| **Condition** | **KS Statistic** | **p-value** |
| --- | --- | --- |
| Consent-GPT | 0.167 | 0.00232 |
| Junior Doctor | 0.272 | 0.00000000722 |
| Treating Surgeon | 0.277 | 0.0000000108 |

*Footnote: KS statistic ranges from 0 to 1, with higher values indicating greater deviation from normality. P-values < 0.05 indicate significant deviation from a normal distribution.*

**Supplementary Note 3.**

*Test for normality of curve in valid consent composite measure.*

The larger the KS statistic, the greater the deviation from a normal distribution. For all three conditions, the p-values are below the significance level of 0.05, indicating significant deviations from normality. This suggests that the valid consent composite measure does not follow a normal distribution in any of the three conditions.

**Supplementary Table S3.**

*One-way ANOVA Results: Effect of Consent Delegate Condition on Perceived Consent Validity.*

| **Source** | **Df** | **Sum Sq** | **Mean Sq** | **F value** | **Pr(>F)** |
| --- | --- | --- | --- | --- | --- |
| Condition | 2 | 10790 | 5395.2 | 19.83 | 6.54E-09 |
| Residuals | 373 | 101482 | 272.1 |  |  |

*Footnote: Df = degrees of freedom, Sum Sq = sum of squares, Mean Sq = mean squares, F value = F-statistic, Pr(>F) = p-value. Significant at p < 0.001.*

**Supplementary Table S4**.

*3x2 Mixed ANOVA Results: Effects of Consent Delegate Condition and Informed Status on Willingness to Sue.*

| **Effect** | **df** | **MSE** | **F** | **ges** | **p value** |
| --- | --- | --- | --- | --- | --- |
| Condition | 2, 373 | 890.8 | 6.23 ** | 0.021 | 0.002 |
| Informed Status | 1, 373 | 507.39 | 873.97 *** | 0.46 | <.001 |
| Condition:Informed Status | 2, 373 | 507.39 | 0.05 | <.001 | 0.955 |

*Footnote: df = degrees of freedom, MSE = mean square error, F = F-statistic, ges = generalized eta-squared (effect size), ** p < 0.01, *** p < 0.001.*

**Supplementary Table S5.**

*Complete Correlation Matrix of All Measured Variables Across Conditions.*

| Variables | Consent-GPT (n=121), r | Junior Doctor (n=131), r | Treating Surgeon (n=124), r |
| --- | --- | --- | --- |
| Valid Consent & Satisfactory Consent | 0.613*** | 0.644*** | 0.735*** |
| Valid Consent & Trust in Delegate | 0.608*** | 0.577*** | 0.629*** |
| Valid Consent & Trust Treating Surgeon | -0.463*** | -0.487*** | *NA* |
| Valid Consent & Share Embarrassing Info | 0.422*** | 0.221* | 0.203* |
| Valid Consent & Sue (Informed) | -0.328*** | -0.382*** | -0.304*** |
| Valid Consent & Sue (Not Informed) | -0.175 | -0.178* | -0.150 |
| Satisfactory Consent & Trust in Delegate | 0.865*** | 0.726*** | 0.804*** |
| Satisfactory Consent & Trust Treating Surgeon | -0.615*** | -0.506*** | *NA* |
| Satisfactory Consent & Share Embarrassing Info | 0.546*** | 0.186* | 0.286** |
| Satisfactory Consent & Sue (Informed) | -0.458*** | -0.225** | -0.349*** |
| Satisfactory Consent & Sue (Not Informed) | -0.224* | -0.238** | -0.083 |
| Trust in Delegate & Trust Treating Surgeon | -0.583*** | -0.599*** | *NA* |
| Trust in Delegate & Share Embarrassing Info | 0.611*** | 0.281** | 0.317*** |
| Trust in Delegate & Sue (Informed) | -0.410*** | -0.271** | -0.300*** |
| Trust in Delegate & Sue (Not Informed) | -0.218* | -0.125 | -0.074 |
| Trust Treating Surgeon & Share Embarrassing Info | -0.491*** | -0.176* | *NA* |
| Trust Treating Surgeon & Sue (Informed) | 0.315*** | 0.341*** | *NA* |
| Trust Treating Surgeon & Sue (Not Informed) | 0.352*** | 0.162 | *NA* |
| Share Embarrassing Info & Sue (Informed) | -0.212* | -0.081 | -0.273** |
| Share Embarrassing Info & Sue (Not Informed) | -0.102 | 0.065 | -0.127 |
| Sue (Informed) & Sue (Not Informed) | 0.136 | 0.344*** | 0.344*** |

*Footnote: This table shows the Pearson correlation coefficients (r) between all variables measured in the study (satisfaction, trust in consent delegate, trust in treating surgeon, comfort sharing information, perceived validity, willingness to sue (informed), and willingness to sue (not informed)) for each condition. Significance levels: * p < 0.05, ** p < 0.01, *** p < 0.001.*
